# Analysing the operative experience of basic surgical trainees in Ireland using a web-based logbook

**DOI:** 10.1186/1472-6920-11-70

**Published:** 2011-09-25

**Authors:** Peter E Lonergan, Jurgen Mulsow, W Arthur Tanner, Oscar Traynor, Sean Tierney

**Affiliations:** 1 National Surgical Training Centre, Colles Institute, Royal College of Surgeons in Ireland, 123 St. Stephen's Green, Dublin 2, Ireland

## Abstract

**Background:**

There is concern about the adequacy of operative exposure in surgical training programmes, in the context of changing work practices. We aimed to quantify the operative exposure of all trainees on the National Basic Surgical Training (BST) programme in Ireland and compare the results with arbitrary training targets.

**Methods:**

Retrospective analysis of data obtained from a web-based logbook (http://www.elogbook.org) for all general surgery and orthopaedic training posts between July 2007 and June 2009.

**Results:**

104 trainees recorded 23,918 operations between two 6-month general surgery posts. The most common general surgery operation performed was simple skin excision with trainees performing an average of 19.7 (± 9.9) over the 2-year training programme. Trainees most frequently assisted with cholecystectomy with an average of 16.0 (± 11.0) per trainee. Comparison of trainee operative experience to arbitrary training targets found that 2-38% of trainees achieved the targets for 9 emergency index operations and 24-90% of trainees achieved the targets for 8 index elective operations. 72 trainees also completed a 6-month post in orthopaedics and recorded 7,551 operations. The most common orthopaedic operation that trainees performed was removal of metal, with an average of 2.90 (± 3.27) per trainee. The most common orthopaedic operation that trainees assisted with was total hip replacement, with an average of 10.46 (± 6.21) per trainee.

**Conclusions:**

A centralised web-based logbook provides valuable data to analyse training programme performance. Analysis of logbooks raises concerns about operative experience at junior trainee level. The provision of adequate operative exposure for trainees should be a key performance indicator for training programmes.

## Background

Considerable concern has been expressed about the adequacy of operative exposure for surgical trainees both in the United Kingdom [1] and the United States [2]. In addition, the provision of surgical training now faces further challenges in the context of working hour restrictions and changing work practices [3]. Recent data from the United States, suggests that the operative exposure of junior surgical trainees may be affected by these changes to an even greater extent [4,5]. Data has been published on the operative experience of senior trainees in general surgery [6,7] and orthopaedics [8-11] in the United Kingdom and Ireland. However, few studies have specifically reported the operative experience of trainees in the formative years of surgical training and those that have been published are based on a subset of operations [12,13] or a single institution [14-16].

Surgical training in Ireland is overseen by the Royal College of Surgeons in Ireland (RCSI) and consists of two phases following internship. Basic surgical training (BST) comprising 2 years of generic surgical training (at the level of senior house officer), where trainees rotate through at least 3 different surgical specialties, followed by competitive entry to 6 years of specialty training in one of 9 specialties. Since July 2007, all new entrants to the BST programme have been required to keep a record of their operative experience using a web-based logbook. This has allowed us a unique opportunity to comprehensively analyse the operative experience of a large cohort of junior surgical trainees and their role in these operations.

The aim of this study was to quantify the operative experience gained by trainees in their first two years of surgical training, post-internship, and to compare their experience to arbitrary training targets. This data may help to identify deficiencies not only in an individual's training, but also within training institutions and the training programme as a whole and ultimately define the need for change in basic surgical training in Ireland in order to maintain the quality of surgical training in the future.

## Methods

The eLogbook (http://www.elogbook.org) is a secure web-based database provided by the Faculty of Health Informatics of the Royal College of Surgeons of Edinburgh. Trainees are provided with individual access to the logbook and it is mandatory for all trainees to record their operative experience using only this logbook. Logbooks are reviewed independently biannually at trainee appraisal sessions and satisfactory completion of the logbook is a requirement for progression through the training programme. Although logbook content is discussed at biannual reviews, there is currently no specific sanction to date for trainees or training programmes that fall short of optimum operative exposure. This study was exempt from review by the RCSI Research Ethics Committee as the data was collected for the purpose of auditing operative exposure by basic surgical trainees. All trainees gave consent for their logbooks to be accessed and analysed.

104 trainees completed the National Basic Surgical Training programme between July 2007 and June 2009. All trainees are required to undertake at least two 6-month posts in general surgery and a minimum of 3 different approved surgical specialties.

Targets for 17 index general surgery operative procedures (9 emergency and 8 elective) were taken from the RCSI Individual Training Plan (ITP) for general surgery (Additional file 1). The ITP contains an arbitrary target number of index elective and emergency operations that a trainee should aim to achieve in a 6-month training post, as specified by the BST Surgical Training Committee of RCSI.

The logbooks for all trainees who commenced the BST programme in July 2007 were interrogated and all operative procedures recorded by trainees were analysed. For the purposes of this study, we analysed the two largest cohorts: general surgery and orthopaedic surgery. Trainees who withdrew from the training programme or did not complete the training programme were excluded.

## Results

### General Surgery

**Figure 1:**
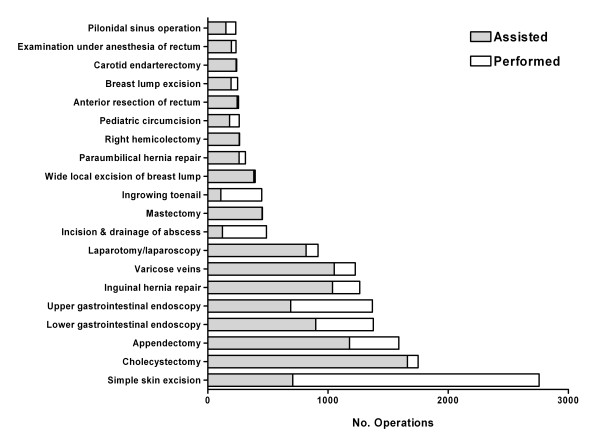
**The 20 most frequently recorded general surgery operations by basic surgical trainees**.

A total of 23,918 general surgery operations were recorded by 104 trainees during the 2-year training programme. The 20 most commonly recorded operations were identified and categorized according to the trainee's role in the operation: either 'assisted' or 'performed' (independently or with senior supervision) (Figure 1). The most commonly recorded general surgery operation was simple skin excision which accounted for 2,757 cases in total. Of the 20 most commonly recorded operations, trainees were the primary operator in the majority of only 3 of these procedures: simple skin excision (2,050 of 2,757; 74.4%), incision and draining of an abscess (366 of 488; 75.0%) and ingrown toenail operation (340 of 448; 75.9%). Trainees were the primary operator in almost half of the upper gastrointestinal endoscopy procedures recorded (679 of 1,369; 49.6%).

In order to put a trainee's operative experience in context, a number of index procedures from the RCSI Individual Training Plan (ITP) for general surgery were identified. Not all procedures from the ITP could be identified from our data, as the logbook permits recording only complete operations. The average number of 17 procedures from the ITP (9 emergency and 8 elective) performed and assisted by trainees were calculated (Table 1). Of the index emergency procedures, trainees were involved in an average of 15.3 (± 10.1) appendectomies; performing an average of 4.0 (± 4.9) and assisting with an average of 11.3 (± 8.2). Trainees performed an average of 2.4 (± 2.5) abscess drainages and assisted with an average of 0.8 (± 1.5).

**Table 1:** The average number of index general surgery operative procedures performed and assisted by basic surgical trainees

	Operation	Assisted	Performed	Total
	Appendectomy	11.3 (8.2)	4.0 (4.9)	15.3 (10.1)
	Abscess drainage	0.8 (1.5)	2.4 (2.5)	3.2 (2.8)
	Right hemicolectomy	2.5 (2.9)	0.0 (0.2)	2.5 (2.9)
	Wound debridement	0.9 (1.9)	0.8 (1.6)	1.7 (2.7)
**Emergency**	Central venous catheter insertion	0.4 (1.5)	0.2 (1.2)	0.7 (1.9)
	Perforated duodenal ulcer repair	0.4 (0.8)	0.0 (0.2)	0.5 (0.8)
	Chest drain insertion	0.1 (0.4)	0.4 (1.0)	0.5 (1.3)
	Femoral embolectomy	0.4 (0.8)	0.0 (0.0)	0.4 (0.8)
	Tracheostomy	0.1 (0.4)	0.1 (0.3)	0.2 (0.5)

	Simple skin excision	6.8 (2.8)	19.7 (9.9)	26.5 (11.0)
	Cholecystectomy	16.0 (11.0)	0.9 (1.6)	16.8 (11.5)
	Upper gastrointestinal endoscopy	6.6 (11.4)	6.5 (9.4)	13.2 (15.9)
**Elective**	Lower gastrointestinal endoscopy	8.6 (15.3)	4.6 (8.0)	13.2 (18.3)
	Inguinal hernia repair	10.0 (7.3)	2.2 (2.7)	12.2 (8.6)
	Ingrown toenail operation	1.0 (1.7)	3.3 (4.6)	4.3 (5.0)
	Pilonidal sinus operation	1.4 (1.9)	0.8 (1.4)	2.2 (2.5)
	Skin graft	0.3 (0.6)	0.1 (0.3)	0.4 (0.8)

Data are expressed as means (± standard deviation) for the number of assisted, performed and total operations for all trainees (n = 104) across the 2 year training program.

Eight elective index procedures were examined, with the most commonly recorded being simple skin excision with an average of 26.5 (± 11.0) per trainee. Of these, trainees performed an average of 19.7 (± 9.9) and assisted with an average of 6.8 (± 2.8). Trainees assisted with an average of 16.0 (± 11.0) cholecystectomies and performed an average of 0.9 (± 1.6). A similar number of upper and lower gastrointestinal endoscopies were recorded, with trainees performing an average of 6.5 (± 9.4) and 4.6 (± 8.0) upper and lower endoscopies respectively. An average of 12.2 (± 8.6) inguinal hernia repairs were recorded with trainees assisting and performing an average of 10.0 (± 7.3) and 2.2 (± 2.7) respectively.

The ITP also specifies an arbitrary target number of emergency and elective operative procedures that a trainee should aim to assist and perform during a 6-month training post and Table 2 shows the percentage of trainees that achieved these targets over the complete 2-year training programme. Of the index emergency procedures, approximately one third of trainees reached the operative target for femoral embolectomy, appendectomy and right hemicolectomy. Twenty-three trainees (22%) recorded performing one or one chest drain insertions. However, only 2 trainees (2%) had reached the required target of central venous catheter insertions. Analysis of the elective procedures, found that 94 (90%) trainees assisted with 5 or more cholecystectomies. Almost two-thirds of trainees met the target for inguinal hernia repair and simple skin excision. Forty-one (39%) and 28 (27%) trainees performed 5 or more upper and lower gastrointestinal endoscopies respectively. Although, not all trainees met the operative target for any one procedure, the percentage of trainees achieving target operative levels was higher in the elective compared with the emergency procedures.

**Table 2:** Operative targets for index general surgery operative procedures

	Operation	Level x Target	Trainees Achieving Target
	Right hemicolectomy	Assisted x3	39 (38)
	Appendectomy	Performed x5	33 (32)
	Femoral embolectomy	Assisted x1	31 (30)
	Abscess drainage	Performed x5	24 (23)
**Emergency**	Chest drain insertion	Performed x1	23 (22)
	Wound debridement	Performed x2	16 (15)
	Perforated duodenal ulcer repair	Assisted x2	13 (13)
	Tracheostomy	Assisted x1	12 (12)
	Central venous catheter insertion	Performed x5	2 (2)

	Cholecystectomy	Assisted x5	94 (90)
	Inguinal hernia repair	Performed x1	67 (64)
	Simple skin excision	Performed x5	64 (62)
**Elective**	Upper gastrointestinal endoscopy	Performed x5	41 (39)
	Pilonidal sinus operation	Performed x1	41 (39)
	Lower gastrointestinal endoscopy	Performed x5	28 (27)
	Ingrown toenail operation	Performed x5	25 (24)
	Skin graft	Assisted x1	25 (24)

This table shows the target number of general surgery operative procedures and the level (assisted or performed) that a trainee is expected to achieve for index operations. The number of trainees who achieved the target number of procedures are expressed as a percentage (in parentheses) of the total trainee cohort (n = 104).

### Orthopaedic Surgery

Between July 2007 and June 2009, 72 (out of 104 in total) BST trainees rotated through a 6-month post in orthopaedic surgery. This cohort recorded a total of 7,551 operations with an average of 104 operations per trainee over the duration of the post.

**Figure 2:**
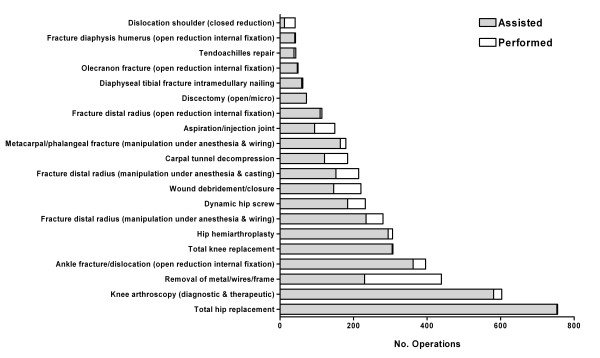
**The 20 most frequently recorded orthopaedic operations by trainees**.

The 20 most frequently recorded operations by the trainee cohort were identified (Figure 2). These operations were categorized according to whether the trainee was the primary operator (independently or with senior supervision), or acted as an assistant. The most commonly recorded operation by trainees was total hip replacement (n = 755), followed by knee arthroscopy (n = 603). Of these 20 operations, trainees performed the greatest proportion of closed reductions of dislocated shoulders (70.7%), followed by removal of metal (47.6%).

The top 15 procedures where the trainee acted as primary operator are listed in Table 3. The most frequently performed operation was removal of metal in which trainees performed an average of 2.9 (± 3.27). Trainees performed an average of 1.03 (± 2.23) debridement/closures of wounds. For each of the remaining procedures, an average of less than 1 operation per trainee was performed during the 6-month training post.

**Table 3:** The 15 most frequently performed orthopaedic operations by basic surgical trainees

Operation	No. Operations Performed	Average per trainee (n = 72)
Removal metal/wires/frame	209	2.90 (3.27)
Wound debridement/closure	74	1.03 (2.23)
Carpal tunnel decompression	63	0.88 (2.03)
Fracture distal radius (manipulation under anaesthesia & casting)	62	0.86 (1.74)
Aspiration/injection joint	55	0.76 (1.15)
Dynamic hip screw	48	0.67 (1.27)
Fracture distal radius (manipulation under anaesthesia & wiring)	46	0.64 (1.29)
Ankle fracture/dislocation (open reduction, internal fixation)	34	0.47 (1.50)
Dislocation shoulder closed reduction	29	0.40 (2.69)
Knee arthroscopy (diagnostic and therapeutic)	22	0.31 (0.56)
Nail bed repair	21	0.29 (1.66)
Metacarpal/phalangeal fracture (manipulation under anaesthesia & wiring)	15	0.21 (0.44)
Hip hemiarthroplasty	12	0.17 (0.41)
Removal foreign body from skin/subcutaneous tissue	11	0.15 (0.75)
Fracture shaft radius (manipulation under anaesthesia & casting)	10	0.14 (0.65)

The 15 most frequently performed orthopaedic operations (either independently or with senior supervision) by trainees and the average per trainee. Data shown are total number of operations and mean (± standard deviation) per trainee.

The 15 operations where trainees assisted a more senior surgeon are shown in Table 4. Trainees assisted with an average of 10.46 (± 6.21) total hip replacements, 8.07 (± 4.90) knee arthroscopies and 5.03 (± 3.93) open reduction internal fixation of ankle fractures. Of the remaining operations, trainees assisted with an average of between 1.00 to 4.24 operations in the 6-month training post.

**Table 4:** The 15 most frequently assisted orthopaedic operations by basic surgical trainees

Operation	No. Operations Assisted	Average per trainee (n = 72)
Total hip replacement	753	10.46 (6.21)
Knee arthroscopy (diagnostic and therapeutic)	581	8.07 (4.90)
Ankle fracture/dislocation (open reduction, internal fixation)	362	5.03 (3.93)
Total knee replacement	305	4.24 (6.22)
Hip hemiarthroplasty	294	4.08 (2.58)
Fracture distal radius (manipulation under anaesthesia & wiring )	234	3.25 (3.48)
Removal metal/wires/frame	230	3.19 (3.99)
Dynamic hip screw	184	2.56 (2.22)
Metacarpal/phalangeal fracture (manipulation under anaesthesia & wiring)	164	2.28 (1.39)
Fracture distal radius (manipulation under anaesthesia & casting)	152	2.11 (3.91)
Wound debridement/closure	146	2.03 (2.33)
Carpal tunnel decompression	121	1.68 (2.64)
Fracture distal radius (open reduction, internal fixation)	109	1.51 (1.67)
Aspiration/injection joint	94	1.31 (1.17)
Discectomy (open/micro)	72	1.00 (4.50)

The 15 operations that trainees most frequently assisted with and the average per trainee. Data shown are total number of operations and mean (± standard deviation) per trainee.

## Discussion

In this study, we have demonstrated that basic surgical trainees are failing to meet modest targets for operative exposure as defined by the national body for surgical training in Ireland. Basic surgical training in Ireland at the time of this study was a 2-year programme comprising four 6-month training posts, of which at least two posts must be in general surgery and was designed to ensure trainees grasp basic surgical principles and are exposed to a range of specialities. To correct for variation in individual posts, we analysed the operative exposure of trainees throughout the full 2-year BST programme but the training targets outlined herein apply to individual (6-month) posts. The operative experience accumulated by the majority of trainees over the two-year period failed to meet the targets for an individual 6-month rotation alone. This clearly highlights a worrying lack of operative exposure amongst basic surgical trainees.

Basic surgical training in Ireland is similar to that in the United Kingdom and shares some features in common with programmes in Europe and the United States [17]. Upon completion of basic surgical training, aspiring surgeons compete for entry into 6 years of specialty surgical training. Recent data from the United States has shown concerning deficiencies in the operative exposure of general surgical trainees upon the completion of training [18]. Similar trends have recently emerged for senior trainees in general surgery [6,7] and orthopaedics [8-11] from the United Kingdom. We have now shown that operative exposure during basic surgical training is sub-optimal. This is of particular importance as there is evidence that work hour restrictions in the United States affect the operative exposure of junior surgical trainees to the greatest extent compared with senior trainees [4,5].

Our data shows, that while trainees may not achieve individual targets for operative procedures, their overall caseload is potentially sufficient to allow them to meet the majority of targets if they were given greater opportunity as the primary operator. Thus, the time spent as the assistant represents a missed opportunity for surgical training for junior trainees. Similar to our findings, analysis of orthopaedic logbook data from the United Kingdom has shown that there are significant missed opportunities for training in the operating room [8,10,11,19] and this effect is greater the more junior the trainee [11]. With challenges in the provision of operative exposure to trainees at all levels, it is possible that procedures traditionally performed by junior trainees have now shifted to more senior trainees.

Logbook data is validated by the individual consultant trainer responsible for the basic surgical trainee; however, we accept that some inaccuracies are likely to be present. It is difficult to quantify this but reports from the literature suggest up to 10% of the data recorded may contain inaccuracies [20] and ensuring completeness of data is likely to be an on-going challenge. Under-reporting of operative procedures may explain the relatively low number of certain common surgical procedures recorded, such as abscess drainage and wound debridement. It has been suggested that trainees under-report cases by up to 20% [21]. Another possible source of inaccuracy may result from inappropriate recording of the trainee's role in the operation, or limitations of the logbook in recording specifically the operation that was ultimately performed. Furthermore, the logbook only records complete operations and does not recognize where the trainee performed specific elements of an operation even though they were not the primary operator. Indeed, a component-based approach to recording operative exposure may be more useful than crudely measuring numbers of procedures, particularly at junior trainee level and has been developed within the logbook for neurosurgical procedures.

The operative targets used in this study are entirely arbitrary and are defined by the national training body as a reasonable minimum requirement for trainees. These targets do not, however, give any indication as to an individual trainees' proficiency or competency and logbook data should be used as part of a global assessment framework rather than in isolation. While confirmed progression to proficiency would be preferable to quantity of procedures in determining progress through training, objective assessment of technical skill and proficiency in surgery is challenging. Logbooks have traditionally acted as a surrogate marker of proficiency, however it is clear that the level of operative exposure required for proficiency will vary greatly amongst individuals. Increasingly it is recognized that the use of surgical simulators allows not only the attainment of skills for commonly performed procedures but also the objective assessment of an individual's proficiency. Attainment of skills in a surgical simulation laboratory can shorten the learning curve and improve performance in the operating room [22-24]. The validation of simulation in surgical training marks a turning point and the potential now exists to train a surgical trainee to a high level of objectively measured skill before they are permitted to operate on a patient. A key aspect of surgical training in the future will be the adoption of a proficiency-based, progression training paradigm that encompasses objective structured assessment of technical skills in the simulation laboratory [25] and traditional logbooks of operative experience.

Work hour restrictions for residents were put in place by the Accreditation Council for Graduate Medical Education (ACGME) in the United States in 2003 (80 hours per week) and by the European Union as apart of the European Working Time Directive (EWTD) in 2009 (48 hours per week). A significant number of reports have examined operative volumes as a result of the restrictions, with conflicting results [26], with published studies demonstrating an improvement [27,28], no change [28-30] or a decrease in operative exposure [4,5,31]. Limited evidence from the United Kingdom suggests that EWTD is adversely affecting trainee operative exposure in general surgery [7,32] and also orthopaedics [9]. This enforced change represents an opportunity to reconsider how surgical training at all levels is delivered to maximize training within the constraints of a shorter working week.

## Conclusions

Going forward, we believe it is essential that a combination of effective use of simulation and adequate operative exposure in the operating room, for trainees at the beginning of their training be given greater attention. This will pose significant challenges in the context of the rapidly changing landscape of surgical training and will require imaginative solutions to increase operative exposure, facilitate skills development and objectively assess technical competence. Our data suggest the overall volume of cases is sufficient to allow individuals to gain adequate experience, but the number of cases spent as the assistant rather than the primary operator represents a missed opportunity for training. It is essential that such opportunities are maximized. We believe that the provision of adequate operative exposure to junior surgical trainees should be a key performance indictor for surgical training programmes. While new technologies and teaching methods may help bridge the gap, it will be only through accurate monitoring of trainee activity in the clinical setting that standards in surgical training can be maintained and future challenges met.

## Competing interests

The authors declare that they have no competing interests.

## Authors' contributions

PEL and JM acquired and analysed the data; and wrote the first draft of the manuscript. ST, WAT and OT designed the study and revised the manuscript. All authors read and approved the final manuscript for publication.

## Authors' information

Peter E Lonergan, MB, BCh, MRCSI completed the National Basic Surgical Training Programme in June 2010 and is currently a research fellow in urology.

Jurgen Mulsow, MD, FRCSI is a trainee on the Higher Surgical Training Programme in general surgery.

W Arthur Tanner, MD, FRCSI is Director of Surgical Affairs at the Royal College of Surgeons in Ireland.

Oscar Traynor, MCh, FRCSI is Director of the National Surgical Training Centre at the Royal College of Surgeons in Ireland and Consultant Surgeon at St. Vincent's University Hospital, Dublin.

Sean Tierney, MCh, FRCSI is Dean of Professional Development and Practice at the Royal College of Surgeons in Ireland and Consultant Surgeon at the Adelaide & Meath Hospital, Dublin.

## Pre-publication history

The pre-publication history for this paper can be accessed here:

http://www.biomedcentral.com/1472-6920/11/70/prepub

## Supplementary Material

Additional file 1 **Target procedures for general surgery posts on the National Basic Surgical Training Programme**. A list of emergency and elective procedures from the Individual Training Plan (ITP) a basic surgical trainee would be expected to either perform or assist with during a 6 month post in general surgery. Click here for file
