# Supplementary material for: Analysing the operative experience of basic surgical trainees in Ireland using a web-based logbook
Source: BMC Med Educ. 2011 Sep 25;11:70. doi: 10.1186/1472-6920-11-70 (PMC3189901; doi:10.1186/1472-6920-11-70)
Supplement: Additional file 1 — Target procedures for general surgery posts on the National Basic Surgical Training Programme. A list of emergency and elective procedures from the Individual Training Plan (ITP) a basic surgical trainee would be expected to either perform or assist with during a 6 month post in general surgery. [file 1472-6920-11-70-S1.DOC]

**Target procedures for general surgery posts on the National Basic Surgical Training Programme**

| Emergency Procedures | | Elective Procedures | |
| --- | --- | --- | --- |
|  | | | |
| Procedure | Level X Number Expected | Procedure | Level X Number Expected |
| Drainage Abscess | P X 5 | Arterial Suturing | A X 3 |
| Appendicectomy | S X 3 | Bowel Anastamosis | A X 5 |
| Central Venous Access | P X 5 | Breast Biopsy | S X 1 |
| Creation of Stoma | A X 2 | Cholecystectomy | A X 5 |
| Wound Debridement | P X 2 | Endoscopy Upper G.I. | S X 5 |
| Femoral Embolectomy | A X 1 | Haemorrhoidectomy | A X 3 |
| Right Hemicolectomy | A X 3 | Injection / RBL piles | P X 3 |
| Laparotomy Opening | S X 5 | Pilonidal Sinus | S X 1 |
| Laparotomy Closing | S X 2 | Ingrown toenail/Nailbed Incision | P X 5 |
| Orchidopexy – Torsion | S X 1 | Inguinal Hernia Repair | S X 1 |
| Bowel Anastomosis | A X 5 | Laparoscopy – create pneumoperitoneum | S X 1 |
| Small Bowel Resection | A X 1 | Excision of sub-Cutaneous swelling | P X 5 |
| Suture bleeding Peptic Ulcer | A X 2 | Excision of skin lesion | P X 5 |
| Suture perforated Peptic Ulcer | A X 2 | Scrotal Exploration | A X 3 |
| Tracheostomy | A X 1 | Split Skin Grafting | A X 1 |
| Catheterisation | P X 5 | Flexible Sigmoidoscopy | S X 5 |
| Chest Drain Insertion | S X 1 | Rigid Sigmoidoscopy | S X 5 |

###### Abbreviations: P, performed (without senior supervision); S, supervised (performed the operation with senior supervision); A, assisted.
